# Antimicrobial Resistance of ESKAPE Pathogens Identified in Patients with Necrotizing Fasciitis: A 10-Year Retrospective Study

**DOI:** 10.3390/medicina62040665

**Published:** 2026-03-31

**Authors:** Mădălina Olivia Radu-Adameşteanu, Elena Rodica Dragu, Bogdan Liviu Chioaru, Ana Cătălina Ţânţu, Corina Daniela Ene, Andrei Creţu, Camelia Andreea Georgescu, Cristian Sorin Hariga

**Affiliations:** 1 Department 11, Discipline Plastic and Reconstructive Surgery, Bucharest Clinical Emergency Hospital, University of Medicine and Pharmacy “Carol Davila”, 050474 Bucharest, Romaniabogdan.chioaru@umfcd.ro (B.L.C.); ana-catalina.tantu0625@rez.umfcd.ro (A.C.Ţ.); andrei.cretu@drd.umfcd.ro (A.C.); andreea-camelia.georgescu@rez.umfcd.ro (C.A.G.);; 2 Clinic of Plastic Surgery, Aesthetic and Reconstructive Microsurgery, Emergency Clinical Hospital Bucharest, 050474 Bucharest, Romania; 3 Department 3, Discipline Internal Medicine and Nephrology—Clinical Nephrology Hospital “Dr. Carol Davila”, University of Medicine and Pharmacy “Carol Davila”, 050474 Bucharest, Romania; corina.ene@umfcd.ro; 4 Clinical Nephrology Hospital “Dr. Carol Davila”, 010731 Bucharest, Romania

**Keywords:** necrotizing fasciitis (NF), ESKAPE pathogens, MDR ESKAPE pathogens

## Abstract

*Background and Objectives*: ESKAPE pathogens—*Enterococcus faecium*, *Staphylococcus aureus*, *Klebsiella pneumoniae*, *Acinetobacter baumannii*, *Pseudomonas aeruginosa*, and *Enterobacter* spp.—are major contributors to antimicrobial resistance and are associated with considerable morbidity and mortality. Their involvement in community-acquired necrotizing fasciitis (CA-NF) remains insufficiently characterized. This study aimed to determine whether ESKAPE pathogens are implicated in cases of CA-NF and to describe their prevalence, antimicrobial resistance profiles, and associated clinical outcomes. *Materials and Methods*: We conducted a retrospective single-center study of NF cases treated in the Plastic Surgery Department of the Emergency Hospital Bucharest (2012–2022). Sixty-five patients met clinical and surgical diagnostic criteria; cases without microbiological data were excluded. Of these, 21 patients had ESKAPE pathogens isolated within 60 min of admission and formed the study cohort. Demographic, clinical, laboratory, microbiological, susceptibility, treatment, and outcome data were analyzed descriptively, with a focus on associated mortality with exploratory comparisons to ESKAPE-negative cases. *Results*: ESKAPE pathogens were identified in 31.8% (21/65) of patients. *S. aureus* was most common (61.9%); 14.3% were *MRSA* (Methicillin resistant *Staphylococcus aureus*) and 47.6% *MSSA* (Methicillin sensitive *Staphylococcus aureus*). Overall, 52% of ESKAPE isolates were multidrug-resistant and 12% were extensively drug-resistant. These resistance patterns have implications for empiric therapy in community-acquired NF. *Conclusions*: ESKAPE pathogens accounted for a substantial proportion of CA-NF and frequently displayed multidrug resistance. These findings highlight the importance of considering ESKAPE pathogens in empiric management strategies for NF, particularly within regional clinical practice.

## 1. Introduction

Infectious diseases remain a major contributor to global mortality and continue to impose a substantial health burden across Europe. According to Eurostat, infectious disease–related deaths in the European Union (EU) have increased by 9% over the past five years, while Romania has experienced a marked increase of 43% during the same period [1].

Necrotizing fasciitis (NF) is a rapidly progressive infection of the skin, fascia, and soft tissues, characterized by extensive tissue necrosis, sepsis, and multiorgan failure, associated with substantial morbidity and mortality, particularly when initial management is delayed. Reported mortality rates range from 25% to 35% [1]. Optimal outcomes depend on early clinical recognition, immediate surgical debridement, and prompt initiation of appropriate antimicrobial therapy.

The global incidence of NF has risen to 0.3–15 cases per 100,000 individuals [2,3,4], driven by population aging, increasing prevalence of chronic comorbidities, widespread use of broad-spectrum antibiotics, and the emergence of highly virulent environmental bacterial strains [5,6,7,8]. During the COVID-19 pandemic, notable shifts in antimicrobial susceptibility patterns among community-acquired pathogens were observed, accompanied by prolonged hospitalizations and increased morbidity and mortality. These trends highlight the need to reassess existing treatment guidelines and clinical practice standards [9,10].

Antimicrobial resistance—identified by the World Health Organization (WHO) as a critical global health threat [11]—is exemplified by the ESKAPE pathogens: *Enterococcus faecium*, *Staphylococcus aureus*, *Klebsiella pneumoniae*, *Acinetobacter baumannii*, *Pseudomonas aeruginosa*, and *Enterobacter* spp. [2,3,9,12,13]. are recognized as major contributors to global antimicrobial resistance and are increasingly implicated in aggressive community-and hospital-acquired infections.

The study was conceived in 2010, during a period of growing international attention to ESKAPE pathogens as leading drivers of antimicrobial resistance [1,2]. However, data from Southeast Europe, and Romania in particular, have remained scarce, especially concerning resistance patterns in CA-NF.

This study investigates the role of ESKAPE pathogens in community-acquired necrotizing fasciitis, outlines their antimicrobial resistance patterns, and assesses alignment with current treatment recommendations to support more targeted antibiotic strategies and improve patient outcomes. It provides the first regional evidence of ESKAPE-associated resistance in necrotizing fasciitis, with the objective of informing more targeted antibiotic strategies and enhancing patient safety and quality of care [14].

## 2. Materials and Methods

### 2.1. Institutional Management Protocol (Clinical Emergency Hospital—Internal Protocols Written by the Department Responsible for Healthcare Associated Infections)

All patients were treated according to the institutional necrotizing fasciitis protocol, which includes:

- Immediate clinical assessment upon presentation;
- Bacteriologic sampling within the first 60 min;
- Surgical debridement initiated within 60 min of diagnosis;
- Early antimicrobial therapy and supportive management for multi-system organ failure (MSOF);
- Daily surgical wound debridement and lavage;
- Repeat bacteriologic testing every five days or sooner if clinical deterioration occurs.

### 2.2. Inclusion and Exclusion Criteria

#### 2.2.1. Inclusion Criteria

- Adults (≥18 years);
- Clinical diagnosis of necrotizing fasciitis;
- Bacteriologic samples obtained within 60 min of presentation;
- Positive identification of ≥1 ESKAPE organism;
- Complete clinical, laboratory, and microbiology datasets;
- Full compliance with the institutional NF management protocol.

#### 2.2.2. Exclusion Criteria

- Healthcare exposure within the preceding 90 days;
- Prior antimicrobial therapy;
- Non-ESKAPE pathogens (e.g., *group A Streptococcus*, anaerobes);
- Suspected healthcare-associated infection;
- Incomplete clinical or microbiological data.

Because the study period spanned an entire decade (2012–2022), antibiotic treatment protocols necessarily varied over time, reflecting updates to national and international clinical guidelines and evolving best practices.

### 2.3. Microbiological Methods

Microbiological sampling was performed within 60 min of emergency room presentation to ensure accurate classification of community-acquired infections and to avoid misclassification of early hospital-acquired cases, particularly for *Acinetobacter baumannii* and *Klebsiella pneumoniae*. Patients with prior healthcare exposure (hospitalization, surgery, dialysis, long-term care, or antibiotics within 90 days) were excluded from the CA-ESKAPE group.

Antimicrobial susceptibility testing followed European Committee on Antimicrobial Susceptibility Testing (EUCAST) methodology. Minimum inhibitory concentration (MIC) and disk diffusion results were interpreted according to EUCAST breakpoints applicable at the time of testing. Because EUCAST breakpoints changed over the 2012–2022 period, isolates were retrospectively reclassified using current Multidrug resistant (MDR), Extreme drug resistant (XDR), and Pan drug resistant (PDR) definitions to ensure consistency.

Resistance phenotypes were assigned according to established criteria

- MDR: Nonsusceptible to ≥1 agent in ≥3 antimicrobial classes;
- XDR: Susceptible to ≤2 antimicrobial classes;
- PDR: Resistant to all tested agents.

Isolates were tested against 11 antimicrobial classes [15,16,17], and complete results were reported without selective omission.

Tested antibiotic classes included β-lactams (penicillins, cephalosporins, carbapenems, monobactams), aminoglycosides, fluoroquinolones, macrolides, sulfonamides, amphenicols, and rifampicin. Last-line antimicrobial agents, including oxazolidinones and glycopeptides, were not routinely incorporated into the susceptibility testing panel. Lipopeptides are not commonly utilized in our country molecular assays for detecting specific resistance determinants—such as erm or mecA—are not available at our institution. Accordingly, the characterization of antimicrobial resistance in this study is based solely on phenotypic susceptibility testing.

Only infections caused by ESKAPE organisms—*Enterococcus faecium*, *Staphylococcus aureus*, *Klebsiella pneumoniae*, *Acinetobacter baumannii*, *Pseudomonas aeruginosa*, and *Enterobacter* spp.—were included. All other pathogens were excluded from our study (*group A Streptococcus*, anaerobic germs).

### 2.4. Data Collection and Variables

Demographic, clinical, laboratory, and microbiology data were extracted and organized using Microsoft Excel 2021. Of the original 65 patients, 44 with non-ESKAPE NF were excluded, leaving 21 patients with confirmed ESKAPE infections (monomicrobial or polymicrobial).

### 2.5. Statistical Analysis

Statistical analyses were performed using IBM SPSS Statistics for Windows, Version 29.0 (IBM Corp.; Armonk, NY, USA). Continuous variables were compared using *t*-tests with significance set at *p* = 0.05 and a 95% confidence interval. Normality of distribution was assessed using the Kolmogorov–Smirnov test. Relationships between variables were evaluated using Spearman’s rank correlation coefficient.

### 2.6. Ethical Approval

The study was conducted according to the principles of the Declaration of Helsinki. Ethical approval was obtained from the Ethics Committee of the Clinical Emergency Hospital Bucharest (No. 4114/28 January 2026). Written informed consent was obtained from all participants or their legal representatives.

## 3. Results

Of the 65 patients admitted with necrotizing fasciitis (NF), 21 (32.3%) had ESKAPE pathogens identified at admission. A total of 25 ESKAPE isolates were recovered from these 21 patients. Patients with non-ESKAPE pathogens were excluded. This study describes the demographic, clinical, laboratory, and bacteriological characteristics of patients with CA-NF caused by ESKAPE organisms.

### 3.1. Microbiological Profile

Bacteriological sampling obtained from purulent discharge, pre-existing wounds, or intraoperative tissue samples was performed within the first hour of arrival, prior to the initiation of empiric antibiotic therapy.

Gram-positive ESKAPE pathogens accounted for 71.4% of necrotizing fasciitis cases, whereas Gram-negative organisms comprised the remaining 28.6%.

The pathogen distribution in our dataset, therefore, represents a different microbiological classification for NF in community-acquired cases involving high-priority, antimicrobial-resistant organisms. Our ESKAPE-focused approach complements—rather than contradicts—existing classifications by characterizing a clinically important, under-recognized subgroup of NF. Specifically, we observed unique patterns of monomicrobial and polymicrobial NF caused exclusively by ESKAPE pathogens in patients without prior healthcare exposure [18,19,20,21,22,23,24,25,26,27,28].

Our dataset reflects a distinct pathogen distribution from the traditional type I/II framework. We classified type one CA-NF as being caused by one single ESKAPE pathogen (monomicrobial), while type II (polymicrobial) CA-NF represents the association of more than one ESKAPE pathogen.

In our study, CA-NF was monomicrobial in 66.7% of cases (14/21) and polymicrobial in 33.3% (7/21). Monomicrobial (single pathogen) infections were dominated by *Staphylococcus aureus* (61.9%). *MRSA* accounted for 14.3% of all S. aureus isolates in the total cohort (3/21) and 33.3% among the 15 isolates included in the resistance analysis.

Acinetobacter baumannii appeared exclusively in polymicrobial CA-NF (multiple ESKAPE pathogens), in contrast with its typical association with healthcare environments (Table 1).

Monomicrobial NF

Among monomicrobial infections (*n* = 14), *Staphylococcus aureus* was the most frequent pathogen (61.9%), followed by *Klebsiella pneumoniae* (14.3%), *Enterococcus faecium* (9.5%), *Pseudomonas aeruginosa* (9.5%), and *Enterobacter* spp. (4.8%). No monomicrobial *Acinetobacter baumannii* infections were identified (Table 2).

Polymicrobial NF

Polymicrobial infections, defined as an association of ESKAPE pathogens, involved most frequently *S. aureus* with *A. baumannii* (28.6%) and *P. aeruginosa*, *Providencia stuartii*, and *Proteus mirabilis* were present in association in 14.3% each, but not described further, due to exclusion criteria of all non-ESKAPE pathogens.

### 3.2. Antimicrobial Resistance Patterns

A total of 27 bacterial strains were isolated from the 21 patients; 25 of these were ESKAPE organisms. Antimicrobial susceptibility testing was performed using standard phenotypic methods according to clinical laboratory guidelines. Resistance was recorded as a binary variable (resistant = 1, susceptible = 0).

Overall, 61.9% of ESKAPE isolates were resistant to ≥2 antibiotic classes. The resistance profile was as follows: MDR 47.6%, XDR 14.3%, and PDR 0%. The remaining 38.1% retained susceptibility to most antibiotic classes.

The reported 52% MDR rate represents the overall proportion of MDR organisms among all ESKAPE pathogens. Resistance categories (MDR/XDR/PDR) were assigned using current international definitions.

*Staphylococcus aureus*

*Staphylococcus aureus* (*n* = 15) was the most prevalent pathogen. Seven isolates (46.7%) were MDR, and two (13%) were XDR—both from polymicrobial NF cases. 14.3% (3/21) were *MRSA* and 47.6% (10/21) were *MSSA*, with *MRSA* confirmed by EUCAST-based phenotypic testing. High resistance rates were observed for penicillin (86.7%), macrolides (60.0%), and clindamycin (46.7%), while susceptibility to aminoglycosides and fluoroquinolones was preserved (Table 3).

Due to its highest frequency in the CA-NF ESKAPE cohort, we considered it important to provide a detailed description of the *Staphylococcus aureus* antibiotic resistance/susceptibility profile, with the intention that this table be used by other authors for comparison.

*Enterococcus faecium*

*E. faecium* was identified in two patients. One isolate was MDR, and one was XDR, showing resistance to all classes except cephalosporins and carbapenems. Both isolates were resistant to fluoroquinolones, macrolides, and rifampicin, with partial resistance to β-lactams. These findings are consistent with advanced resistance phenotypes.

*Klebsiella pneumoniae*

*K. pneumoniae* was found in two patients. One strain was MDR, with resistance to penicillins, cephalosporins, and fluoroquinolones. Both strains remained susceptible to macrolides, carbapenems, sulfonamides, chloramphenicol, and rifamycin. Resistance to third- and fourth-generation cephalosporins suggests ESBL (Extended spectrum beta-lactamase) production.

*Acinetobacter baumannii*

*Acinetobacter baumannii* was isolated exclusively in polymicrobial NF (two cases). Both strains were MDR and resistant to piperacillin/tazobactam, ceftazidime, cefepime, cefotaxime, aminoglycosides, fluoroquinolones, and carbapenems. Susceptibility remained for chloramphenicol, rifampicin, macrolides, cefuroxime, ceftriaxone, and most penicillins.

*Pseudomonas aeruginosa*

*P. aeruginosa* was isolated in two monomicrobial cases and one polymicrobial infection. Resistance was heterogeneous. Notably, 66.7% of isolates were resistant to ceftriaxone, piperacillin/tazobactam, cefepime, and imipenem. Resistance to meropenem, ertapenem, aminoglycosides, fluoroquinolones, ceftazidime, and penicillins ranged from 33.3% to 66.7%. All isolates were sensitive to ampicillin, amoxicillin, ampicillin/sulbactam, cefuroxime, macrolides, rifampicin, chloramphenicol, and trimethoprim–sulfamethoxazole.

*Enterobacter* spp.

*Enterobacter* spp. was isolated in one monomicrobial case. The strain exhibited resistance to penicillins and cefuroxime but retained susceptibility to the remaining antibiotic classes. We found it relevant to show the overall resistance patterns for all identified ESKAPE pathogens identified in CA-NF, included in the present study. Our study describes a relevant 10% of NF cases treated in Romania in 2012–2022. We felt the need to create a summarized version of antibiotic resistance patterns for the ESKAPE pathogens included in the study because the scientific literature mainly highlights case reports in our country. In Southeastern Europe, scientific data is scarce; studies from Hungary and Serbia confirm that ESKAPE pathogens became a major health problem, with increased incidence and prevalence.

**Figure 1:**
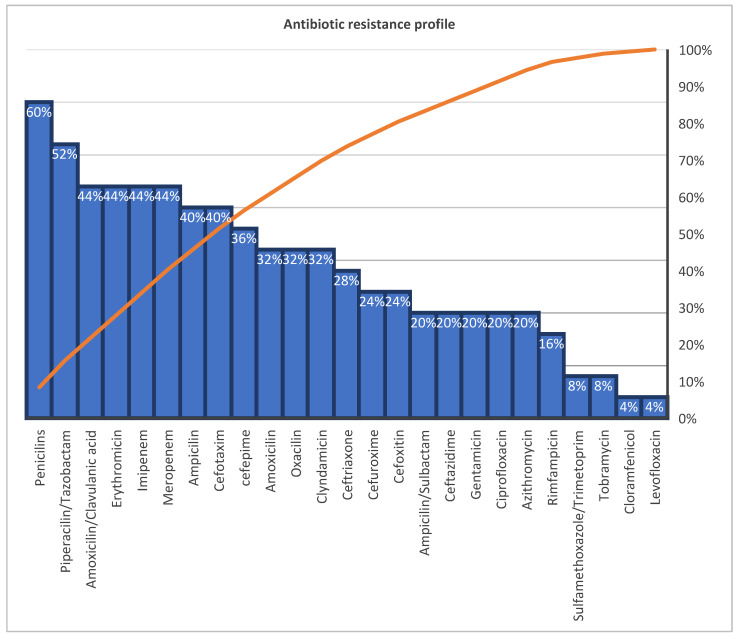
Antibiotic resistance profile of CA-NF ESKAPE pathogens.

Overall, antibiotic resistance patterns are summarized in Figure 1.

Based on the statistical analysis of the results, we created a resistance pattern table for the ESKAPE pathogens and the tested classes of antibiotics. We did not include in the tested antibiotic classes the last line antibiotics (oxazolidinones, Lincosamides, glycopeptides, polymixines). According to our protocols, these are not routinely tested, but are used as a last resort treatment. Lipopeptides are not commonly used in our clinic.

The comparative antimicrobial resistance among CA-NF ESKAPE pathogens is detailed in Table 4 and Table 5.

Main resistance features for each ESKAPE pathogen to common antibiotic classes are presented in Table 5.

## 4. Discussion

Necrotizing fasciitis (NF) is a rare yet life-threatening soft-tissue infection. Early administration of broad-spectrum antimicrobial therapy, together with prompt and radical surgical debridement, remains essential for improving patient outcomes [1]. Due to its low incidence, most published reports on NF are limited by small sample sizes. For example, a 2024 cohort study from Germany involving 42 patients identified *Staphylococcus aureus* as the predominant pathogen, with no ESKAPE organisms or MDR/XDR/PDR strains detected [3].

More recent studies from India (2026), using similar methodology, reported that ESKAPE pathogens were present in 61.6% (*n* = 1311) of soft-tissue infections, with S. aureus being the most frequently isolated organism (59.5%) and a high prevalence of MRSA (52.2%). Gram-negative pathogens displayed MDR profiles in 15.3% of cases [12].

In Southeastern Europe, scientific evidence regarding NF remains limited. A retrospective three-year study from Serbia identified 13 NF cases, reporting pathogen distributions like our findings, with *Staphylococcus aureus*, *Klebsiella pneumoniae*, and *Pseudomonas aeruginosa* as the most frequently isolated organisms [9].

In Hungary, ESKAPE pathogens have shown a marked increase in prevalence over the past decade, particularly *K. pneumoniae* and *P. aeruginosa*, while *Enterococcus faecium* and *Acinetobacter baumannii* accounted for most MDR cases [29].

In Romania, available scientific evidence on NF consists primarily of isolated case reports, with none addressing patterns of bacterial resistance [4]. Regarding ESKAPE pathogens more broadly, a retrospective analysis from the intensive care unit of Sibiu County found that 75.6% (*n* = 160) of hospital-acquired isolates belonged to the ESKAPE group, predominantly *K. pneumoniae*, *A. baumannii*, and *P. aeruginosa* [5]. These findings align with our results, supporting the observation of a high prevalence of ESKAPE pathogens in our region.

A recent investigation from an infectious disease hospital in Southeastern Romania further highlights the regional burden of antimicrobial resistance. An analysis of 4293 bacterial isolates demonstrated substantial prevalence of MDR ESKAPE pathogens, with the highest resistance rates observed in *MRSA* (86.6%), *Acinetobacter baumannii* (36.8%), *Pseudomonas aeruginosa* (29.1%), and *Klebsiella pneumoniae* (24.4%). These findings underscore the significant circulation of multidrug-resistant organisms in Romanian healthcare settings and reinforce the relevance of our own results [6].

This study highlights the critical role of ESKAPE pathogens in community-acquired necrotizing fasciitis (NF), underscoring the substantial therapeutic challenges posed by widespread antimicrobial resistance.

Across all ESKAPE isolates recovered from NF patients, high rates of resistance were observed—particularly among Gram-negative organisms. Multidrug resistance (MDR) was identified in every ESKAPE species, with *Acinetobacter baumannii* demonstrating the most severe resistance profile, including universal carbapenem resistance. Notably, both patients infected with *A. baumannii* experienced 100% mortality. Gram-positive pathogens also contributed significantly to the resistance burden: *Staphylococcus aureus* exhibited high resistance rates, including a 14.3% prevalence of MRSA, while Enterococcus faecium displayed resistance to several first-line agents, thereby limiting empiric therapeutic options.

The presence of *MRSA*, ESBL-producing *Klebsiella pneumoniae*, carbapenem-resistant *Acinetobacter baumannii*, and MDR *Pseudomonas aeruginosa* confirms that narrow-spectrum empiric regimens are insufficient in the management of CA-NF. Infections caused by community-acquired resistant ESKAPE organisms prompt revision of empiric antibiotic guidelines in NF and require escalation to broad-spectrum or combination therapy. Delays in appropriate antimicrobial coverage contribute to increased morbidity, the need for repeated surgical debridement, and higher mortality rates.

At present, no universally accepted antimicrobial guideline exists for NF; consequently, treatment regimens vary across institutions and national protocols. Over the 10-year study period, antibiotic protocols at our center were continuously adapted in response to evolving resistance patterns. No included patient reported allergies to penicillin or other antimicrobial agents. Current empiric regimens at our institution depend on clinical presentation. In our clinic, soft-tissue infections are typically treated with amoxicillin/clavulanic acid (resistance rate 44%) combined with sulfamethoxazole/trimethoprim (resistance 8%), fluoroquinolones (levofloxacin 4%; ciprofloxacin 20%), and metronidazole.

Based on our study, it seems that misdiagnosed NF as a simple soft-tissue infection, typically treated with the above-mentioned protocol, has a better outcome than a more aggressive association of antibiotics.

In cases of multiple organ system failure (MSOF) or clinical suspicion of NF, empiric therapy includes vancomycin or linezolid combined with piperacillin/tazobactam (resistance 52%) or a carbapenem (imipenem resistance 44%). Clindamycin (resistance 32%) is added when septic shock is suspected. These findings prompt an urgent revision of current antibiotic therapy guidelines in NF, at least for our region. The continuous use of reserved antibiotics has led to a failure in treatment with an increased resistance pattern, especially in community-acquired cases.

Despite these challenges, several antimicrobial agents retained favorable susceptibility profiles among the ESKAPE isolates. High sensitivity rates were observed for rifampicin (84%), sulfamethoxazole/trimethoprim and tobramycin (92%), and chloramphenicol and levofloxacin (96%). These agents may serve as useful adjuncts in targeted therapy once susceptibility data become available.

The severe resistance patterns observed in *Acinetobacter baumannii* are particularly concerning. Carbapenem-resistant Acinetobacter (CRAB) is associated with poor outcomes in NF and other necrotizing soft-tissue infections due to limited therapeutic options and high virulence. Similarly, the significant burden of *MRSA* supports routine empiric inclusion of anti-*MRSA* therapy in suspected NF. The cephalosporin-resistance patterns of *Klebsiella* and *Enterobacter* isolates—compatible with ESBL and AmpC phenotypes—reinforce the importance of early recognition of resistant organisms and timely escalation to advanced agents such as carbapenems, β-lactam/β-lactamase inhibitor combinations, or novel antimicrobial agents where available.

Based on our statistical analysis using Pearson correlation (significance threshold *p* < 0.05), several clinically relevant associations were observed. In this cohort of 21 patients, a moderate negative correlation was found between pathogen type and MDR phenotype (r = −0.524, *p* = 0.018). XDR infection was significantly associated with increased mortality (r = 0.509, *p* = 0.022). Coinfection correlated positively with both XDR status (r = 0.454, *p* = 0.044) and mortality (r = 0.485, *p* = 0.026). No associations could be evaluated for PDR due to a lack of variability. Given the relatively small sample size, these findings should be interpreted cautiously and considered hypothesis-generating (Table 6).

Strengths and Limitations

The strengths of our study are listed below:

- 10% of all NF cases in our country in a ten-year period;
- Focus on ESKAPE pathogens, the highest-risk organisms in severe, rapidly progressive infections;
- Specific focus on CA-NF;
- Detailed, antibiotic-level resistance profiling across all recovered isolates;
- Direct clinical relevance to necrotizing fasciitis, a time-critical surgical emergency;
- Clear implications for improving empiric antimicrobial strategies in high-severity cases;
- The first study on CA-NF with ESKAPE pathogens in Romania is one of the few in southeastern Europe.

The limitations are listed below

- Small sample sizes for certain pathogens limit subgroup generalizability;
- Absence of molecular resistance characterization (e.g., PCR or whole-genome sequencing);
- Antimicrobial resistance patterns do not include all antimicrobial classes (oxazolidinones, lincosamides, glycopeptides, polymixines).

These limitations are inherent to the study of rare, life-threatening infections such as necrotizing fasciitis and do not diminish the clinical relevance of antimicrobial resistance surveillance in this population.

Taken together, the findings underscore the importance of:

- Aggressive and timely surgical debridement;
- Rapid microbiological identification with prompt antibiogram testing;
- Early initiation of broad-spectrum empiric therapy, followed by targeted de-escalation.

These elements remain essential to the effective management of necrotizing fasciitis caused by ESKAPE pathogens.

## 5. Conclusions

Necrotizing fasciitis is a rare but rapidly progressive and life-threatening infection in which early broad-spectrum antimicrobial therapy and prompt radical surgical debridement remain critical to improving survival. Because of the low incidence of NF, most published studies—both globally and in Europe—are limited by small cohorts, contributing to significant gaps in understanding its evolving microbiological and resistance patterns.

International data consistently identify *Staphylococcus aureus* as the predominant pathogen, with variable involvement of ESKAPE organisms and multidrug-resistant strains. Although studies from Germany and Serbia reported lower proportions of MDR pathogens, recent findings from India, Hungary, and Romania demonstrate a rising prevalence of MDR and XDR organisms in severe soft-tissue infections, reflecting an alarming global trend.

In Romania, scientific data on NF remain scarce and largely restricted to case reports. However, existing national surveillance studies reveal a substantial burden of ESKAPE pathogens and high antimicrobial resistance rates in hospital-acquired infections. These findings corroborate the context in which our study was conducted and highlight the urgent need for region-specific epidemiological data.

Our cohort of 65 NF patients provides important new insights into the bacteriological landscape of this condition in Romania. ESKAPE pathogens were identified in 31.8% of cases—a significant proportion for a rare disease—demonstrating that multidrug-resistant organisms are no longer confined to healthcare settings but increasingly appear in community-acquired infections. Among all isolates, *Staphylococcus aureus* remained the most frequent pathogen, while monomicrobial infections predominated. Resistance to penicillins reached 60%, and resistance to β-lactams, macrolides, and carbapenems approached 40%. In contrast, the highest susceptibility was observed for levofloxacin, chloramphenicol, tobramycin, sulfamethoxazole/trimethoprim, and rifampicin.

These results underscore the need for cautious selection of empiric antimicrobial therapy, particularly when using penicillins, cephalosporins, macrolides, or carbapenems in regions with high MDR prevalence. Until species identification and susceptibility results are available, broad-spectrum coverage remains essential. As always, immediate and thorough surgical debridement continues to be the cornerstone of NF management.

Although limited by sample size, this study provides novel and clinically relevant data on antimicrobial resistance in NF within a region where evidence is scarce. Our findings highlight the growing role of ESKAPE pathogens in severe soft-tissue infections and reinforce the importance of continuous surveillance to guide empiric therapy and improve patient outcomes.

## Figures and Tables

**Table 1:** CA-NF classification monomicrobial CA-NF 66.7% versus polymicrobial CA-NF 33.3%.

CA-NF Classification	N	%
Monomicrobial CA-NF	14	66.7%
Polymicrobial CA-NF	7	33.3%

**Table 2:** Distribution of ESKAPE pathogens in monomicrobial NF.

Pathogen	*n*	%
*Staphylococcus aureus*	13	61.9%
*Klebsiella pneumoniae*	3	14.3%
*Enterococcus faecium*	2	9.5%
*Pseudomonas aeruginosa*	2	9.5%
*Enterobacter* spp.	1	4.8%

**Table 3:** Antimicrobial resistance profile of *Staphylococcus aureus* isolates (*n* = 15).

Antibiotic Class	*Antibiotic*	Resistant Isolates (%)
Penicillins	Ampicillin	40.0%
	Penicillin	86.7%
	Amoxicillin	40.0%
	Oxacillin (MRSA)	14.3%
β-lactam/β-lactamase inhibitors	Amoxicillin/clavulanic acid	46.7%
	Ampicillin/sulbactam	20.0%
	Piperacillin/tazobactam	46.7%
Cephalosporins	Cefuroxime	26.7%
	Ceftriaxone	26.7%
	Ceftazidime	20.0%
	Cefepime	33.3%
	Cefotaxime	0%
	Cefoxitin	14.3%
Aminoglycosides	Gentamicin	0%
	Amikacin	0%
	Tobramycin	0%
Fluoroquinolones	Ciprofloxacin	0%
Macrolides	Azithromycin	33.3%
	Erythromycin	60.0%
Lincosamides	Clindamycin	46.7%
Carbapenems	Meropenem	20.0%
	Imipenem	46.7%
	Ertapenem	13.3%
Sulfonamides	Trimethoprim–sulfamethoxazole	6.7%
Amphenicols	Chloramphenicol	6.7%
Rifamycins	Rifampicin	13.3%

**Table 4:** Antibiotic resistance profile of ESKAPE pathogens.

*Pathogen*	*n*	*High β-Lactam Resistance*	*Cephalosporin Resistance*	*Carbapenem Resistance*	*Fluoroquinolone Resistance*	*Macrolides Resistance*	*Lincosamides Resistance*	*MDR Phenotype*
*E. faecium*	2	Moderate	Low	None	High	High	None	Yes
*S. aureus*	15	Very high	Moderate	Moderate	Low	High	Moderate	Yes
*Klebsiella* spp.	2	High	High	None	Moderate	None	None	Yes
*Acinetobacter* spp.	2	Very high	Very high	Very high	Very high	None	None	Yes (CRAB)
*P. aeruginosa*	*3*	Moderate	Moderate	Moderate	Moderate	None	Moderate	Yes
*Enterobacter* spp.	1	High	High	None	Low	None	None	Possible

**Table 5:** Comparative antimicrobial resistance patterns among ESKAPE pathogens isolated from CA-NF cases.

Pathogen	*n*	Main Resistance Features	MDR Present
*Enterococcus faecium*	2	Fluoroquinolones, macrolides, rifampicin	Yes
*Staphylococcus aureus*	15	Penicillins, macrolides; 14.3% MRSA	Yes
*Klebsiella* spp.	3	Penicillins, cephalosporins (ESBL pattern)	Yes
*Acinetobacter baumannii*	3	β-lactams, carbapenems	Yes
*Pseudomonas aeruginosa*	4	β-lactams, fluoroquinolones, carbapenems	Yes
*Enterobacter* spp.	2	Penicillins, cephalosporins (AmpC)	Possible

**Table 6:** Correlations between CA-NF ESKAPE germ resistance and deaths.

Parameters	Death (N = 21 Subjects)	
	r	*p*
Pathogens	0.042	0.857
Coinfection	0.485	0.026
MDR (at least one agent in 3 antimicrobial categories)	0.167	0.482
XDR (non-susceptibility to at least one agent in all but two or fewer antimicrobial categories)	0.068	0.776

Strengths. Statistical significance (*p*); Correlation coefficient (r).

## Data Availability

The database used in this study can be found in the hospital informatic system and archives and is not available for public access.

## Abbreviations

The following abbreviations are used in this manuscript:

ESKAPE	*Enterococcus faecium*, *Staphylococcus aureus*, *Klebsiella pneumoniae*, *Acinetobacter baumannii*, *Pseudomonas aeruginosa* and *Enterobacter* spp.
CA	Community acquired
NF	Necrotizing fasciitis
MDR	Multidrug resistant
XDR	Extreme drug resistance
PDR	Pan drug resistance
EU	European Union
WHO	World Health Organization
MRSA	Methicillin resistant Staphylococcus aureus
MSSA	Methicillin sensitive Staphylococcus aureus
ESBL	Extended spectrum beta-lactamase
MSOF	Multiple system and organ failure
